# Spinal cord injury and spinal fracture in patients with ankylosing spondylitis

**DOI:** 10.1186/s12873-022-00635-3

**Published:** 2022-05-02

**Authors:** Po-Hsun Tu, Zhuo-Hao Liu, Mun-Chun Yeap, Yu-Tse Liu, Ying-Ching Li, Yin-Cheng Huang, Tzu-Min Lin, Ching-Chang Chen

**Affiliations:** 1 Department of Neurosurgery, Chang Gung Memorial Hospital at Linkou, Chang Gung Medical College and University, Taoyuan, Taiwan; 2 Department of Internal Medicine, Division of Rheumatology, Immunology and Allergy, Taipei Medical University Hospital, Taipei, Taiwan; 3 Department of Internal Medicine, Division of Allergy, Immunology and Rheumatology, School of Medicine, College of Medicine, Taipei Medical University, Taipei, Taiwan

**Keywords:** Spinal trauma, Spinal fracture, Spinal cord injury, Ankylosing spondylitis, Bamboo spine

## Abstract

**Background:**

Spinal cord injury (SCI) and spinal fracture are major complications in patients with ankylosing spondylitis (AS) who sustain spinal trauma. The purpose of this study was to investigate the incidence, predictors, and sequelae of spinal trauma in patients with AS.

**Methods:**

This retrospective study included patients with AS who were admitted for spinal trauma between January 1, 2006, and June 30, 2016. The study compared clinical outcomes of patients between group 1: SCI alone, group 2: spinal fracture alone (no SCI), and group 3: both SCI and spinal fracture.

**Results:**

Of the 6285 patients with AS admitted during the retrospective study period, only 105 suffered from spinal trauma and were enrolled in the study. Case number in group 1, 2, and 3 was 11(10.48%), 45(42.85%), and 49(46.67%), respectively. Among the patients with spinal fractures, 52.1% had SCI. Bamboo spine was significantly more prevalent in the fracture group than in the nonfracture group (78.7% vs. 36.4%; *P* = 0.006). Patients with SCI had more instances of subluxation or dislocation (48.3% vs. 8.9%; *P* < 0.001) and more cases of spinal epidural hematoma (SEH; 21.7% vs. 2.2%; *P* = 0.003) than patients without SCI. The rate of delayed diagnosis for spinal fracture was 31.4%, with one-third of patients developing delayed SCI. Among the patients with incomplete SCI, 58.3% achieved neurological improvement after treatment (*P* = 0.004).

**Conclusions:**

Patients with AS and bamboo spine at radiograph had a higher rate of spinal fracture, which may be an important factor in SCI in patients with AS. Spinal fractures involving the C3–C7 region, subluxation or dislocation, severe spinal fracture, and SEH were found to be predictive of SCI, and SCI in patients with AS resulted in higher mortality and complication rates.

## Background

Ankylosing spondylitis (AS) is a chronic inflammatory disease that mainly affects the axial skeleton. AS is characterized by progressive bone loss, erosion, and syndesmophyte formation, all of which lead to progressive spinal rigidity and altered spinal biomechanics [1]. AS thus increases the risk of vertebral fractures from even minor injuries [2–4]. Patients with AS generally have marginal syndesmophyte formation, which presents as the classic “bamboo spine” in radiographic examinations [5]. An ankylosed spine is fragile due to the secondary osteoporosis and loss of mobility that accompany the disease [6], and the risk of fracture increases as the disease progresses over time. A severely ankylosed spine is more susceptible to injury and is relatively unstable compared with a normal spine [2, 7–9]. However, delayed diagnosis of spinal fractures in patients with AS after minor trauma is common. Patients with delayed diagnosis generally present with chronic pain, progressive neurologic deficits, and worsening spinal deformity [10–12].

Spinal trauma includes spinal cord injury (SCI), spinal fracture, or both.[1, 4, 11, 13, 14] Spinal trauma in the ankylosed spine greatly affects morbidity and mortality [3, 11, 13]. The mean prevalence of AS per 10,000 people has been found to be 23.8 in Europe, 16.7 in Asia, 31.9 in North America, 10.2 in Latin America, and 7.4 in Africa [15]. The reported prevalence of spinal fracture in patients with AS varied greatly between Europe and North America, with prevalences of 10% and 17%, respectively [6, 8, 16, 17], and rates of SCI of 19% and 91%, respectively [4, 11, 16, 18, 19]. Because AS is an uncommon disease, data on the predictors of this condition are scarce [4, 13], especially in Asia. The purpose of this study was to investigate the incidence, predictors, and sequelae of spinal trauma in patients with AS at a single tertiary center in Asia.

## Material and methods

### Patient sample

Between January 1, 2006, and June 30, 2016, 6285 patients were treated for spinal fractures or SCI following a spinal injury at our institution. Of the 6285, only 110 patients with AS with spinal trauma were identified. The patients’ charts and images were reviewed by 3 experienced neurosurgeons. A rheumatologist confirmed the diagnosis of AS based on the criteria of the Assessment of Spondyloarthritis International Society [20–23], and 105 patients who had a minimum 2-year follow-up or who died over the course of follow-up were included. This study excluded 4 patients who had a follow-up of less than 1 year and 1 patient for whom key image data were missing. This study was approved by the institution’s institutional review board (IRB No.: 201700858B0).

### Variables

Predefined and generally accepted parameters (listed in Table 1) were used to extract data from electronic medical records, including patient age at the time of injury, patient sex, the initial neurological grading on the American Spinal Injury Association Impairment Scale (AIS), the presence of high-energy trauma [24], the presence of bamboo spine, the presence of subluxation or dislocation, the presence and level of SCI, the presence and level of spinal fracture, the presence and level of spinal epidural hematoma (SEH), the fracture classification, the treatment administered, the outcome at discharge and 2 years after trauma, and the presence and type of complications.

**Table 1:** Definition of parameters in the article

**Low-energy trauma**: not high-energy trauma, such as high-speed traffic accidence or fall > 15 feet [13]
**Delayed diagnosis** (spinal fracture or SCI diagnosis after the day of trauma, less than 24 h count 0 day) **Patient’s delay**: the patient visits a physician after the day of trauma **Doctor’s delay**: the patient was not diagnosed by doctor
**Bamboo spine:** diagnosis by radiologist’s report
**Subluxation:** defined as more than 2 milli-meter distance between the inferior endplate of the neighboring superior vertebra and the superior endplate of the neighboring inferior vertebra at the anterior longitudinal ligament line or dislocation at spinal fracture or spinal cord injury level
**Spinal cord injury and level, spinal fracture and level, spinal epidural hematoma and level** (levels C0–C2, C3–C7, T1–T12, L1–L5)
**Spinal epidural hematoma**: spinal epidural hematoma detected on initial or subsequent computed tomography and/or magnetic resonance imaging
**Fracture classification**
**C0–C2** Atlas fractures classified according to Levine and Edwards [25], fractures of the odontoid process according to Anderson and D’Alonzo [26], and fractures of the odontoid body according to Levine and Edwards [27]
**C3–L5** Fractures classified according to an algorithm derived from the AO Spine fracture classification [28]
**Discharge outcome**, 2 years** after trauma outcome**: AIS
**Complications:** all events associated with treatment and associated with SCI occurring within 2 years after the trauma
**Treatment associated:** instrumentation failure, such as migration or loosening of screws/rods; wound infection
**Spine or spinal cord injury associated:** respiratory failure; pneumonia; pulmonary embolism; pneumothorax; decubitus ulcer; urinary tract infection; sepsis

### Outcome measures

The degree of SCI was manually graded for each patient according to the AIS at the time of the initial SCI diagnosis, at the time of discharge, and at the 2-year follow-up. Patients with a decline in AIS grade of 1 or more (such as a decline from E to D) at any point after injury were considered patients with SCI. We categorized SCI as either complete (AIS grade A) or incomplete (AIS grades B–D). Patients with SCI who demonstrated an improvement of at least 1 AIS grade (such as from D to E) during follow-up were considered neurologically improved.

### Statistical methods

Data were presented as frequency and percentage for continuous variables and as mean and standard deviation for categorical variables. The patients included in the study were divided into 3 groups (group 1: SCI alone, group 2: spinal fracture alone (no SCI), and group 3: both SCI and spinal fracture). The characteristics of patients among the groups were compared using one-way analysis of variance for continuous variables or Fisher’s exact test for categorical variables. The improvement in AIS grade from the time of the first symptoms to the last follow-up in the patients with SCI was tested using the McNemar test. All tests were 2-tailed, and *P* < 0.05 was considered statistically significant. No adjustment for multiple testing (multiplicity) was made in this study. Data analyses were conducted using SPSS 25 (IBM SPSS, Chicago, Illinois).

## Results

**Fig. 1:**
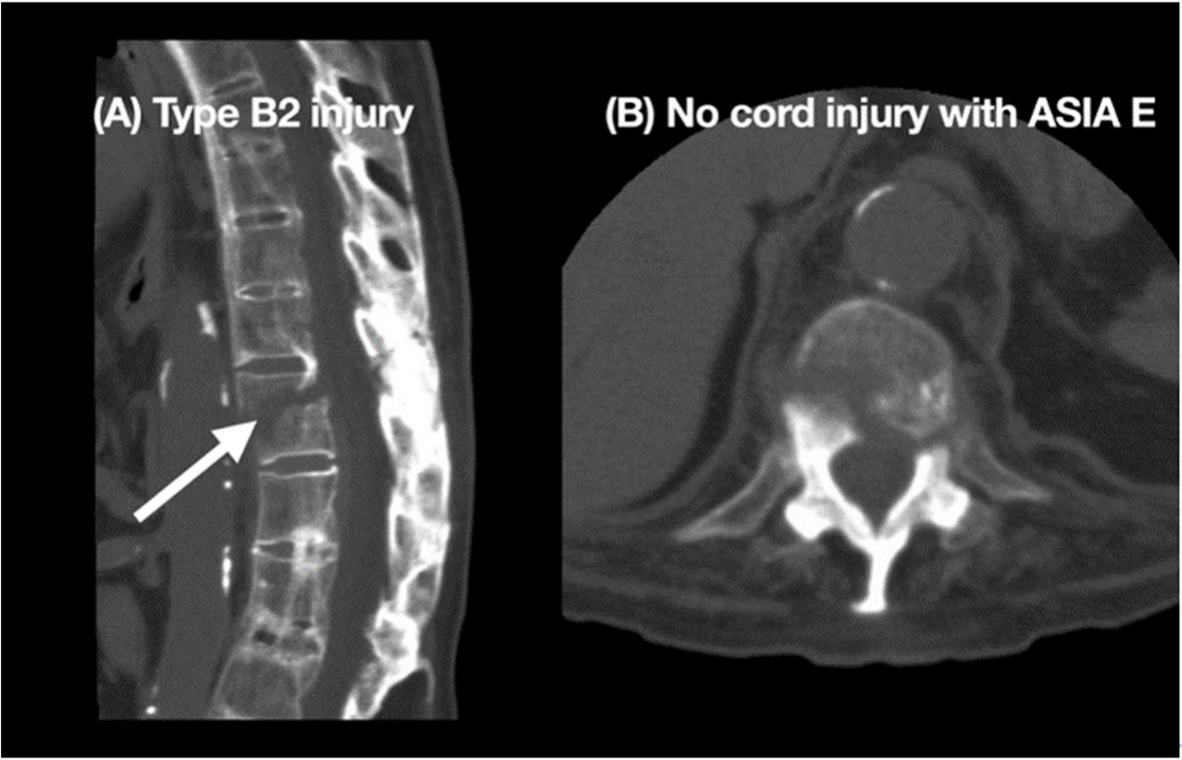
Demonstration of a patient in group 2 (spinal fracture alone). A 63-year-old man with AS presented to the hospital after a falling down accident, without neurological deficit (ASIA Grade E). CT showed a Type B2 fracture (**A**) and the fracture with mild displacement of T11 vertebral body and the fracture line involving and left pedicle (**B**). The patient underwent internal fixation as schedule

**Fig. 2:**
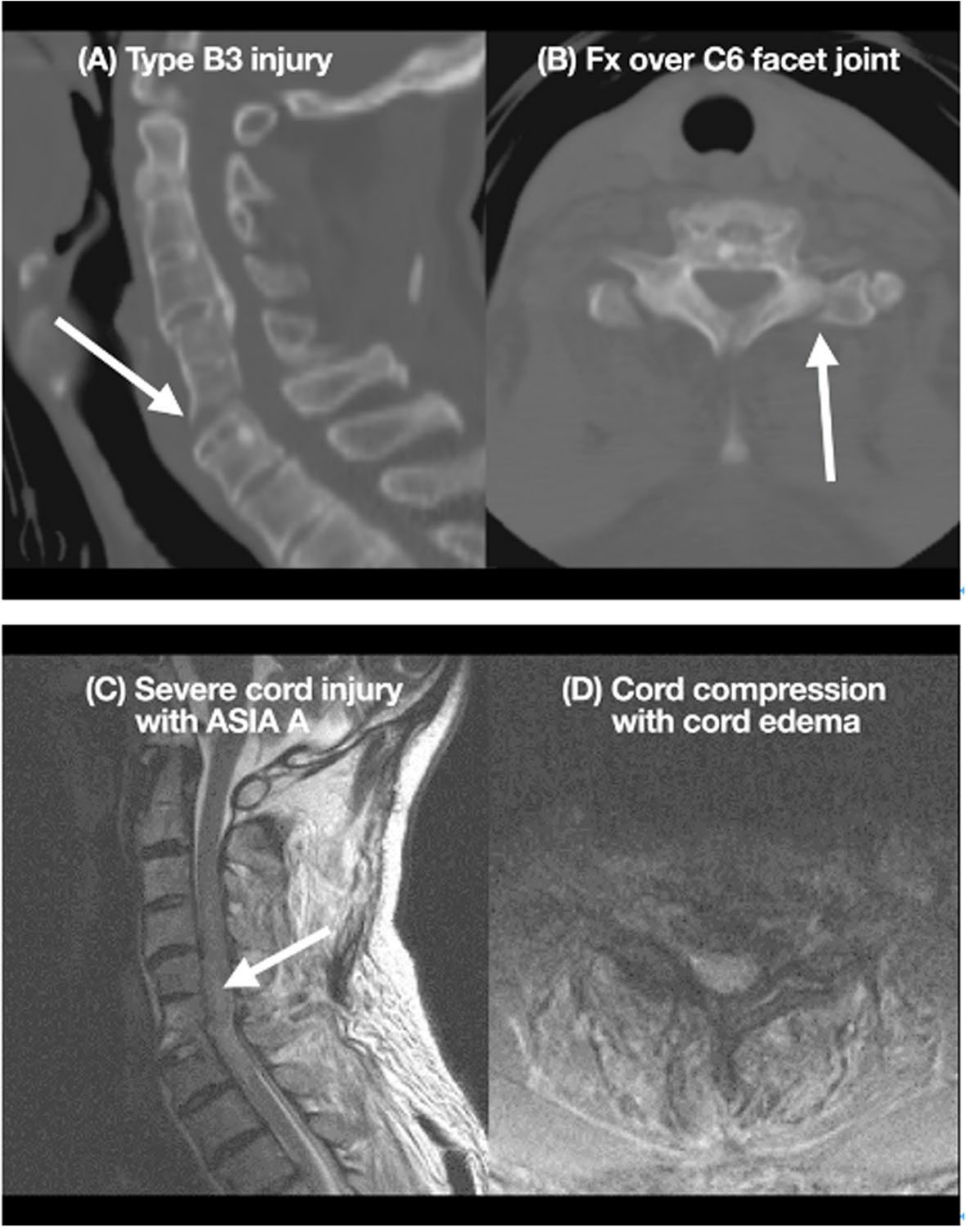
Demonstration of a patient in group 3 (both spinal fracture and cord injury). A 52-year-old man with AS presented to the hospital after a high-energy trauma, with neurological deficit (ASIA Grade A). CT showed a Type B3 fracture (**A**) and the fracture involving C5–6 and left facet (**B**). cervical MRI showed severe cord compression with cord edema (**C**.**D**). The patient underwent reduction, internal fixation and decompression immediately

Of the 105 patients included in the study, 11 patients (10.48%) were in group 1, 45 patients (42.85%) in group 2 (Fig. 1), and 49 (46.67%) in group 3 (Fig. 2). A small percentage of the patients were female (4.8%; 5/105). Fifty-three patients (50.5%) had low-energy trauma alone, 33 (31.4%) had subluxation or dislocation, and 94 (89.5%) had spinal fractures. Spinal fracture involving the C3–C7 region occurred most frequently in patients with both SCI and spinal fracture (65.3%), whereas the thoracic spine was the most frequently fractured region in patients without SCI (44.4%). Of the 105 patients, 60 (57.1%) had SCI, including 12 with complete SCI. Of the 12 cases of complete SCI, C3–7 SCI accounted for 43.8%. Forty-nine patients (52.1%) had both SCI and spinal fracture, and 78 patients (74.3%) presented with bamboo spine in X-ray images (Table 2).

**Table 2:** Characteristics in patients with ankylosing spondylitis according to the spinal cord injury with or without fracture

Variables	Whole cohort (*N* = 105)	Group1: SCI alone (*n* = 11)	Group 2: Fracture alone (*n* = 45)	Group 3: SCI and fracture (*n* = 49)	*P* value
Male	100 (95.2)	10 (90.9)	42 (93.3)	48 (98.0)	0.312
Age (year)	56.2 ± 12.3	55.9 ± 6.2	55.9 ± 15.3	56.6 ± 10.2	0.968
Low-energy trauma	53 (50.5)	6 (54.5)	24 (53.3)	23 (46.9)	0.814
Subluxation or dislocation	33 (31.4)	1 (9.1)^a^	4 (8.9)^a^	28 (57.1)	< 0.001
Location of spinal fractures					< 0.001
None	11 (10.5)	11 (100.0)^a^	0 (0.0)^b^	0 (0.0)	
C0-2	5 (4.8)	0 (0.0)	4 (8.9)	1 (2.0)	
C3-7	37 (35.2)	0 (0.0)^a^	5 (11.1)^a^	32 (65.3)	
T	28 (26.7)	0 (0.0)	20 (44.4)^ab^	8 (16.3)	
L	15 (14.3)	0 (0.0)	14 (31.1)^ab^	1 (2.0)	
Multiple regions involved	9 (8.6)	0 (0.0)	2 (4.4)	7 (14.3)	
Spinal fracture classification					< 0.001
None	11 (10.5)	11 (100.0)^a^	0 (0.0)^b^	0 (0.0)	
A01-A4	13 (12.4)	0 (0.0)	11 (24.4)^a^	2 (4.1)	
B1-B3	42 (40.0)	0 (0.0)^a^	18 (40.0)^b^	24 (49.0)	
C	36 (34.3)	0 (0.0)^a^	13 (28.9)^b^	23 (46.9)	
Others	3 (2.9)	0 (0.0)	3 (6.7)	0 (0.0)	
Location of SEH					0.006
None	91 (86.7)	11 (100.0)	44 (97.8)^a^	36 (73.5)	
C	8 (7.6)	0 (0.0)	0 (0.0)^a^	8 (16.3)	
C-T	4 (3.8)	0 (0.0)	0 (0.0)	4 (8.2)	
L, T-L	2 (1.9)	0 (0.0)	1 (2.2)	1 (2.0)	
Bamboo spine	78 (74.3)	4 (36.4)^a^	31 (68.9)^ab^	43 (87.8)	0.001
Delayed diagnosis	33 (31.4)	0 (0.0)	21 (46.7)^ab^	12 (24.5)	0.003
Delayed diagnosis type (*n* = 33)					1.000
Doctor	6 (18.2)	0 (0.0)	4 (19.0)	2 (16.7)	
Patient	27 (81.8)	0 (0.0)	17 (81.0)	10 (83.3)	
Delayed diagnosis days (*n* = 33)	19.8 ± 20.4	-	26.0 ± 22.5	9.1 ± 9.2	-
SEH	14 (13.3)	0 (0.0)	1 (2.2)^a^	13 (26.5)	0.001
Complication	32 (30.5)	1 (9.1)^a^	6 (13.3)^a^	25 (51.0)	< 0.001
Complication, infection	24 (22.9)	1 (9.1)^a^	3 (6.7)^a^	20 (40.8)	< 0.001

*SCI* spinal cord injury, *SEH* spinal epidural hematoma

Values are given as frequency (%) or mean ± standard deviation

‘^a^’ and ‘^b^’ indicate the value reached a significant difference versus the “SCI and fracture” and “SCI alone” groups, respectively

Delayed diagnosis was made in 33 patients (31.4%). Among these patients, all had spinal fracture; 6 of the patients were placed in the “doctor delay” group. Two of the 6 patients developed SCI at 5 days, and another 2 of the 6 developed SCI at 15 days after the trauma. The remaining 27 patients were placed in the “patient delay” group; 9 of the patients developed delayed SCI within 2 to 90 days after the trauma. The average time between trauma and a confirmed diagnosis for all the patients was 19.8 days (SD = 20.4 days), with a range of 2 to 90 days (Table 2).

Of the 105 patients in this study, 32 received surgical treatment for spinal fracture alone at 13 ± 93.7 days after trauma, and 45 patients received surgical treatment for SCI that consisted of decompression, open reduction, and fixation at 1.8 ± 3.2 days. Of the patients with both SCI and spinal fracture, 6, including 3 patients with AIS grade A, received closed reduction and halo-jacket fixation; 3 patients with mild SCI (initial AIS grade: D) with mild spinal fracture (AO spine fracture classification: A) received conservative treatment. Thirteen patients with mild spinal fractures (AO spine fracture classification: A) without SCI received conservative treatment. Of the 11 patients with SCI alone, 10 were cases of central cord syndrome, and they achieved recovery to AIS grade E; 1 case was a severe head injury with consciousness no recovery to clear. One, 4, 2, 3, and 1 injuries occurred at C3, C4, C5, C6, and C7, respectively. Anterior microdiscectomy with interbody fusion and fixation was performed in 2 cases; the 8 patients with mild SCI (initial AIS grade: D) and the 1 who initially presented with a deep coma received conservative treatment.

SEH was observed in 14 patients (13.3%). Over the course of follow-up, 32 patients (30.5%) experienced complications, with infection being the most common (22.9%). Four patients (3.8%) died during the 2-year follow-up period; 3 of these deaths were due to pneumonia-related septic shock, and 1 was due to pulmonary embolism and cardiac failure with acute respiratory distress syndrome (Table 2).

Among the SCI patients, the AIS grade significantly improved from the time of the first symptoms to the follow-up at 2 years (*P* = 0.004). Among all 60 patients with SCI, 35 achieved an improvement in AIS grade. However, the AIS grades in 4 patients worsened due to the patients who died (*n* = 4) having been given an AIS grade A at the 2-year follow-up (Table 3). Excluding patients with mild SCI (initial AIS grade: D) who did not undergo surgery, 53.3% of the 45 patients who received surgical treatment experienced long-term neurological recovery.

**Table 3:** AIS grade improvement from first symptoms to the follow-up after 2 years in patients with spinal cord injury and received surgical treatment (*n* = 45)

	AIS grade after two years				
Initial AIS grade	A	B	C	D	E
A	9				
B	1	3	3	3	
C	1		4	7	1
D	1			2	10

*AIS* American Spinal Injury Association (ASIA) Impairment Scale

The *P* value of the McNemar test was 0.004

Patients who died were included into “A” group at AIS grade during follow-up period

For patients with and without spinal fracture, bamboo spine appeared significantly more frequently in the fracture group (78.7% vs. 36.4%; *P* = 0.006). Demographic and clinical characteristics were not significantly different, however, between the fracture and nonfracture groups or between the SCI and non-SCI groups. The results revealed that compared with the non-SCI group, more cases involving subluxation or dislocation (48.3% vs. 8.9%; *P* < 0.001), spinal fractures involving the C3–C7 region (53.3% vs. 11.1%; *P* < 0.001), spinal fracture in general (*P* < 0.001), and SEH occurred in the SCI group (21.7% vs. 2.2%; *P* = 0.003; Table 4).

**Table 4:** Baseline characteristics in patients with ankylosing spondylitis according to spinal cord injury or spinal fracture

	Spinal cord injury			Spinal fracture		
Variables	SCI (*n* = 60)	Non-SCI (*n* = 45)	*P* value	Fracture (*n* = 94)	Non-fracture ( *n* = 11)	*P* value
Subluxation or dislocation	29 (48.3)	4 (8.9)	< 0.001	32 (34.0)	1 (9.1)	0.167
Location of spinal fractures			< 0.001			-
None	11 (18.3)^a^	0 (0.0)		-	-	
C0-2	1 (1.7)	4 (8.9)		-	-	
C3-7	32 (53.3)^a^	5 (11.1)		-	-	
T	8 (13.3)^a^	20 (44.4)		-	-	
L	1 (1.7)^a^	14 (31.1)		-	-	
Multiple regions involved	7 (11.7)	2 (4.4)		-	-	
Spinal cord injury level			-			0.001
None	-	-		45 (47.9)^a^	0 (0.0)	
C1-2	-	-		3 (3.2)	0 (0.0)	
C3-7	-	-		35 (37.2)^a^	11 (100.0)	
T	-	-		11 (11.7)	0 (0.0)	
Bamboo spine	47 (78.3)	31 (68.9)	0.367	74 (78.7)	4 (36.4)	0.006
SEH	13 (21.7)	1 (2.2)	0.003	14 (14.9)	0 (0.0)	0.353

*SCI* spinal cord injury, *SEH* spinal epidural hematoma

Values are given as number (%) or mean ± standard deviation

‘^a^’ indicates the value reached a significant difference between the two proportions in the row

## Discussion

Among patients with AS, SCI is a major complication regardless of spinal fracture occurrence. Our results revealed that the SCI rate after spinal trauma in patients with AS was 57.1% (60/105) and the SCI rate in cases also involving spinal fractures was 52.1% (49/94), which are similar to the rates in Europe and North America, which range from 19.7% to 67.2% [3, 4, 11, 13, 14, 18]. Variation in the rate of SCI after spinal fractures in patients with AS may be due to differences in medical referral standards, severity of trauma, and severity of AS.

Diagnoses of fractures in patients with AS are frequently delayed at a rate of 17.1% to 65.4%[2, 4, 11, 14] due to the frequent presence of chronic pain in patients with AS, even in the absence of trauma. Therefore, aggravating pain following minor trauma may be overlooked. Due to alterations in bone density resulting from AS, radiological assessment of fracture in patients with AS can be difficult, with fractures difficult to identify and easily misinterpreted, especially in cases involving fractures at the thoracic spine and thoracolumbar junction. In our study, the rate of delayed diagnosis of spinal fracture was 31.4% (33/105), with 69.7% (23/33) of those cases involving fractures between the T8 and L1 vertebrae. Delay in 6 of the 33 cases was attributed to oversight by a doctor, and delay in the remaining cases was due to patients delaying visits. Oversight may put patients with AS at higher risk of delayed SCI. In our study, one-third of the 33 patients with a delayed diagnosis of spinal fracture developed delayed SCI. Within the initial posttrauma period, 90.9% of these patients had axial pain, such as neck or back pain, and 33.3% had limb numbness. Attending physicians should remain aware of the consequences of delayed diagnosis in patients with AS, even in cases of low-energy trauma. We suggest routine radiographic examination for all patients with AS after trauma and additional computed tomography imaging if axial pain progresses. Magnetic resonance imaging is a viable option for assessing spinal cord injuries and for detecting potential occult fractures [12, 29–31].

Bamboo spine is a radiographic feature in AS that occurs as a result of vertebral body fusion by marginal syndesmophytes. The resulting radiographic appearance is of radiopaque spicules that completely bridge the adjoining vertebral bodies. In our study, 74.3% (78/105) of patients with AS exhibited this feature. We also observed that the patients with bamboo spine had a higher rate of spinal fracture than those without bamboo spine (*P* = 0.006; Table 4). However, the results revealed no significant correlation between bamboo spine and SCI (*P* = 0.367; Table 4). This contradicted our finding that AS with spinal fracture was significantly related to SCI (*P* < 0.001). To clarify this discrepancy, we analyzed the relationship between bamboo spine and spinal fracture in the 60 patients with SCI. Four of the 11 patients who had SCI without spinal fracture presented with bamboo spine, and 43 of the 49 patients with both SCI and spinal fracture had bamboo spine. A comparison of these 2 groups revealed a significant difference (*P* = 0.001; Table 2). Patients with noncomplex compression fractures with intact posterior ligamentous complex (PLC) (AOSpine—Spine Trauma Classification System type A) [32] had a lower rate of SCI than those with complex fractures, which include tension band injuries in cervical spine, and distraction injuries in thoracolumbar spine (type B), or translation injuries (type C) (*P* < 0.001). Subluxation or dislocation was also a risk factor for patients with AS developing SCI (*P* < 0.001). The severity of disruption to the spinal structure is, thus, predictive of SCI in patients with AS. In cases involving a complex fracture, SCI is not only caused by the damage from direct impact but also by further compression from bone fragments, hematoma, or disk material [33]. Therefore, we hypothesize that patients with AS with bamboo spine have a high probability of experiencing spinal fracture. Mild fractures, however, do not necessarily cause SCI in patients with AS.

No universal guidelines have been developed for the management of spinal trauma in patients with AS [31]. Nonoperative treatment, including bed rest, skeletal traction, bracing, or immobilization with a halo-vest, has long been recommended for nondisplaced or minimally displaced fractures of ankylosed spines [31, 34, 35]. However, the inherent instability of these fractures and their high potential for acute displacement may cause severe damage [36]. Therefore, surgical fixation with long segmental instrumentation combined with fusion is recommended [36]. Furthermore, the compression of neurological elements often requires surgical evacuation. Recent studies have demonstrated a trend of higher complication rates in nonoperative patients—for instance, finding higher rates of pulmonary complications and a risk of neurological deterioration [3, 4, 35]. Surgical stabilization usually includes anterior, posterior, or combined fixation, often accompanied by decompression with laminectomy and several osteotomy techniques for deformity correction [37, 38]. In our study, 3 patients with an initial AIS grade A received closed reduction and halo-jacket fixation, and all 3 patients (100%) experienced complications: 1 patient experienced screw loosening and 2 developed pneumonia. By contrast, 7 of 9 patients (77.8%) with an initial AIS grade A experienced complications after surgery, suggesting a trend of lower complication rates in severe SCI.

All 14 patients with SEH developed SCI; SCI thus became a key predictive factor for SCI (*P* = 0.003). SEH occurred in 13.3% of the patients with AS, which is a much higher rate than that of the general population (range: 0.5% to 7.5%) [39, 40]. Compared with fractures that cause SCI immediately, SEH may lead to subacute SCI hours after trauma. The mechanisms underpinning cervical SEH formation are not fully understood; disruption of the posterior longitudinal ligament and spinal epidural vessel rupture, however, may play a vital role [25, 40]. Symptomatic SEH is generally considered a neurosurgical emergency. In the general population, better long-term neurological recovery has been noted after early surgical intervention. However, no major case study has reported the effects of early surgical intervention in patients with AS. In our study, the patients with SCI received surgical treatment 1.8 ± 3.2 days after trauma. Several of these patients had delayed surgical treatment due to delayed diagnosis, old age, comorbidity, or polytrauma that required additional treatments.

None of the patients who had complete SCI at admission exhibited improvement in AIS grade after 2 years. By contrast, 58.3% (35/60) of patients in the incomplete SCI group demonstrated improvement. Overall, patients with AS with incomplete SCI had better long-term neurological recovery. Complication rates were significantly higher (*P* = 0.001) in patients with SCI. The 4 patients who died within 1 year were significantly older (with an average age of 69.2 years). This echoes previous findings of higher mortality in older patients with AS after spinal fracture [11, 13]. Of the patients with AS with different complexities of fracture and varying degrees of SCI in our study, more than 53% of patients achieved long-term neurological recovery after surgical treatment.

### Limitation

This is a single-center study thus obviating patient selection bias. Besides, most subjects achieved long-term follow-up. Meanwhile, this study was limited for its retrospective design, as well as the non-feasibility of analysis on cervical, thoracic and lumbar cohorts separately due to insufficient case number. Thus, further well-designed prospective randomized studies are needed.

## Conclusions

SCI is a major complication of spinal trauma in patients with AS. After spinal trauma, patients with AS with bamboo spine have relatively high rates of spinal fracture. Cervical fracture involving the C3–C7 region, subluxation or dislocation, high severity, and SEH are predictive of SCI. In patients with AS, even low-energy trauma has the potential to cause spinal trauma, including SCI or fracture. Although the prevalence of AS differs across continents, the rate and predictors of SCI after spinal trauma in AS exhibit no obvious differences. Delayed diagnosis of fracture and SCI occur in approximately 30% and 10% of cases of spinal trauma, respectively. Therefore, attending physicians should be aware of their potential effects on patients with AS.

## Data Availability

The datasets used and analyzed during the current study are available from the corresponding author on reasonable request.

## Abbreviations

SCI: Spinal cord injury
AS: Ankylosing spondylitis
SEH: Spinal epidural hematoma
ASAS: Assessment of Spondyloarthritis International Society
AIS: American Spinal Injury Association Impairment Scale
